# The Ontario Animal Health Network: enhancing disease surveillance and information sharing through integrative data sharing and management

**DOI:** 10.1177/10406387211003910

**Published:** 2021-03-25

**Authors:** Cynthia Miltenburg, Tim Pasma, Kathleen Todd, Melanie Barham, Alison Moore

**Affiliations:** Ontario Ministry of Agriculture, Food and Rural Affairs, Guelph, Ontario, Canada; Ontario Ministry of Agriculture, Food and Rural Affairs, Guelph, Ontario, Canada; Animal Health Laboratory, University of Guelph, Ontario, Canada; Animal Health Laboratory, University of Guelph, Ontario, Canada; Ontario Ministry of Agriculture, Food and Rural Affairs, Guelph, Ontario, Canada

**Keywords:** animal diseases, data analysis, data quality, disease management, emerging infectious disease, laboratory diagnosis, Ontario, population health, population surveillance, veterinary pathology

## Abstract

The Ontario Animal Health Network (OAHN) is an innovative disease surveillance program created to enhance preparedness, early detection, and response to animal disease in Ontario. Laboratory data and, where available, abattoir condemnation data and clinical observations submitted by practicing veterinarians form the core of regular discussions of the species-sector networks. Each network is comprised of government veterinarians or specialists, epidemiologists, pathologists, university species specialists, industry stakeholders, and practicing veterinarians, as appropriate. Laboratorians provide data for diseases of interest as determined by the individual network, and network members provide analysis and context for the large volume of information. Networks assess data for disease trends and the emergence of new clinical syndromes, as well as generate information on the health and disease status for each sector in the province. Members assess data validity and quality, which may be limited by multiple factors. Interpretation of laboratory tests and antimicrobial resistance trends without available clinical histories can be challenging. Extrapolation of disease incidence or risk from laboratory submissions to broader species populations must be done with caution. Disease information is communicated in a variety of media to inform veterinary and agricultural sectors of regional disease risks. Through network engagement, information gaps have been addressed, such as educational initiatives to improve sample submissions and enhance diagnostic outcomes, and the development of applied network-driven research. These diverse network initiatives, developed after careful assessment of laboratory and other data, demonstrate that novel approaches to analysis and interpretation can result in a variety of disease risk mitigation actions.

## Introduction

Disease surveillance in animal populations through analysis of veterinary laboratory data is becoming more important than ever before. Focused examination and analysis of animal disease incidence data are used worldwide to help protect national food security by demonstrating the health status of animal populations and freedom from disease. The use of these data may also enable prompt intervention in disease outbreak situations and reveal knowledge or information gaps in order to provide more relevant health information to veterinarians, livestock producers, and companion animal owners. The Ontario Animal Health Network (OAHN), a provincial disease surveillance network, was created to accomplish these objectives in Ontario, Canada.

We provide here a description of OAHN, its structure and function, as well as the types of data streams, including laboratory data, that are integrated for analyses to enhance disease surveillance and information exchange with various stakeholders. Furthermore, several activities and projects that have been developed by OAHN as a result of animal health surveillance in Ontario are highlighted.

### Development and structure of the Ontario Animal Health Network

**Figure 1.**
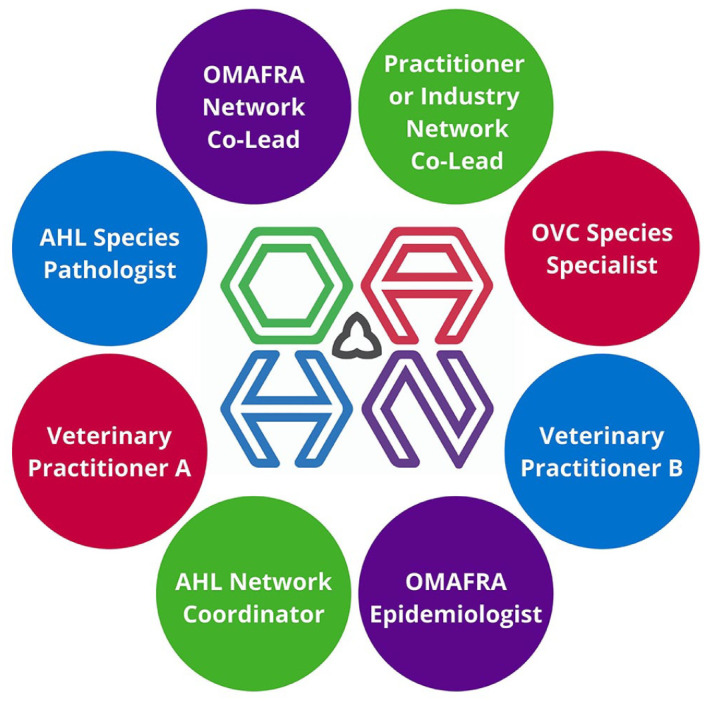
Network members for the Ontario Animal Health Network (OAHN) species-sector networks. Some networks also include members from other government or industry organizations. AHL = University of Guelph Animal Health Laboratory; OMAFRA = Ontario Ministry of Agriculture, Food and Rural Affairs; OVC = Ontario Veterinary College.

Created in 2013 to improve animal health and disease surveillance in the province and modeled after Réseau d’alerte et d’information zoosanitaire (RAIZO; Quebec, Canada), the OAHN is a joint initiative of the University of Guelph Animal Health Laboratory (AHL) and the Ontario Ministry of Agriculture, Food and Rural Affairs (OMAFRA). This disease surveillance network consists of 10 species-sector networks (aquatic animals, bees, bovine, companion animal, equine, poultry, small ruminant, swine, wildlife, and alternative species), and is a collaborative way to evaluate animal health. Each network is jointly led by a government veterinarian or species specialist from OMAFRA and a veterinarian or individual from private practice or industry. Other network members include species specialists from the Ontario Veterinary College (OVC) and other Ontario universities, veterinary anatomic and clinical pathologists from the AHL, an epidemiologist from OMAFRA, up to 4 veterinarians from private practice, and a network coordinator (Fig. 1). Some networks, specifically the swine, bee, and aquatic animal networks, include industry representatives from their species sector.

**Figure 2.**
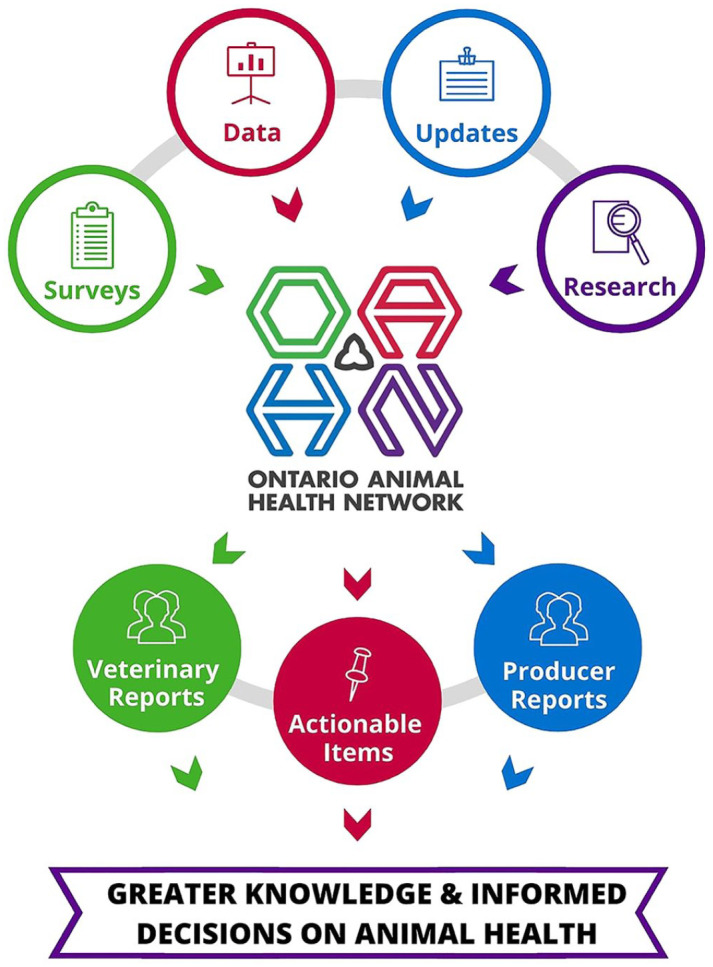
Individual species networks meet to discuss and interpret all sources of information to the networks, including laboratory data, and to discuss implications for animal health, which are incorporated into veterinary and industry reports, research projects, and educational materials produced by the Ontario Animal Health Network.

The networks meet regularly (either quarterly or semi-annually) to discuss clinical impressions from the members, and to review a variety of data sources, including observations provided via survey from a larger number of veterinarians in the field and laboratory data from the AHL (Fig. 2). Laboratory data are also shared from other laboratories for some species: Gallant Custom Laboratories (CEVA) contributes data to the swine network, and Idexx Laboratories Canada contributes data to the equine network. The swine network has chosen to increase membership of industry partners in their network meetings and, as such, a representative from Gallant Custom Laboratories participates in the swine network, contributing to and benefitting from the analysis and discussions of the network.

Condemnation data from Ontario’s provincial abattoirs is provided by OMAFRA for the swine, poultry, small ruminant, and bovine sectors, and condemnation data from federal abattoirs is shared with the poultry and swine networks. OMAFRA also provides aggregate data on provincially regulated pests and diseases of honeybees gathered through inspections as part of the provincial apiary program. Clinical observations from Ontario’s equine, companion animal, swine, and poultry veterinarians are gathered via surveys (Qualtrics) every 3 mo, to provide perspective from private practitioners working in those species sectors. Networks may also utilize an “emerging threats report,” a compilation of recent articles globally sourced from industry reports, news, ProMED (International Society for Infectious Diseases), and the Centre for Emerging and Zoonotic Disease (CEZD; Canadian Food Inspection Agency, Government of Canada), which summarizes global infectious disease and animal health issues of interest to the network.

Networks vary in how they evaluate health data for their species. For a variety of reasons, such as low numbers of practicing veterinarians for that species, a majority of diseases relating to management factors, or a paucity of test result data for the species, not all networks utilize veterinary surveys or analyze laboratory or condemnation data. Even when limited data are available, networks find great benefit for their sectors in regular discussions of pathology observations, government perspectives, as well as industry group observations and concerns. Various organizations are involved in these discussions, including the Canadian Wildlife Health Cooperative, the Canadian Food Inspection Agency, The Department of Fisheries and Oceans Canada, and the Ontario Ministry of Natural Resources and Forestry.

As a result of the process of disease monitoring and network discussion and engagement, each network has the option to pursue research related to disease investigation. Short-term, 12-mo research investigations may be proposed to the network group by any member if further investigation is warranted into a disease or issue specific to that species. Some of these projects serve to address apparent information gaps, such as deficiencies in the dataset or testing available to investigate a disease or syndrome. Other projects may address animal health and welfare educational needs relevant to the sector. These initiatives vary widely in topic and scope, but all offer additional innovative methods to assist with disease response.

For all OAHN species networks, the regular discussions provide a platform for information sharing, gap identification, and idea or research generation that did not exist previously. Each network produces regular products for veterinarians and industry summarizing findings and discussion. Various formats are used to disseminate the information including infographics, podcasts, video, and written reports. This improved communication among involved parties results in increased awareness of disease risks and unique opportunities for each sector and should be recognized as an important facet of disease surveillance. In addition, the close collaboration of government, specialists, industry representatives, and others is an important feature of the OAHN framework. This collaboration results in the identification of species priorities in diseases and important dialogue derived from various backgrounds dedicated to animal health.

### Extraction and analysis of laboratory data

When the networks were established initially, members discussed and selected diseases of interest to the network for monitoring, including diseases with serious health consequences or of economic importance. Network members in the AHL extract pathology, laboratory test, and antimicrobial susceptibility data for individual species networks using existing database queries and new queries designed to obtain specific data for emerging conditions (BusinessObjects XI; SAP). AHL pathologists review the query reports to provide further analysis and interpretation.

Participating private laboratories also provide data quarterly. Data contributions from the various laboratories are assessed separately; case counts and test results are not amalgamated given the variations in test types and methodology. Data requirements and partners have evolved over time for each network. Data are reviewed and assessed primarily through a descriptive approach. Counts of positive laboratory tests are summed by quarter for specific pathogens, such as *Salmonella* spp. in cattle or influenza A virus in swine, and may be further broken down by serovar or subtype. Pathology diagnoses are categorized by system or clinical problem that prompted the submission or are summarized by select key diagnoses. Pathologists may follow up with submitting veterinarians on cases of interest, such as new or unusual disease conditions or clinical presentations.

Non-statistical methods are used to analyze data. Cases and test results in the current quarter are compared to the number in the previous quarter and occasionally to the same quarter in the previous year. Trends are monitored by viewing data in tabular or graphical form. Unusual cases are highlighted and brought to the attention of the network. Networks may also monitor conditions in which an idiopathic or no diagnosis is captured, given that these may indicate an emerging disease. The monitoring of submissions with “diagnosis not reached” was proposed as an important method of early detection for new and emerging diseases.^8^

Statistics on condemnations from provincially licensed abattoirs, compiled on the OMAFRA website, are also reviewed by applicable networks. Condemnation data from both bovine and swine abattoirs in Ontario were shown as potentially useful for disease surveillance.^1,17^ Given the large number of conditions and dispositions for animals, networks tend to select and monitor key categories or conditions.

### Laboratory data quality and validation

Although the use of laboratory data helps triangulate other sources of data such as network surveys, challenges exist with analyzing large volumes of laboratory data. Reviewing and collating cases retrieved from a laboratory information management system requires a significant time commitment, particularly for pathology cases. The use of pathology diagnostic codes can assist with the rapid collation of cases, and a “case summary” system was recently implemented, making the process of case review and data organization more efficient.

Laboratory data must be interpreted carefully because of submission bias, whereby cases submitted for laboratory testing may not reflect cases seen in the general population.^2^ The cases that veterinarians choose, and that producers consent to proceed with laboratory testing may represent the most severe or unusual cases. It is possible that other cases of the disease exist in the population, but these may have less severe signs or better response to empirical treatment. Submission bias may also be present among data summarized for antimicrobial susceptibility surveillance.^6^ Infections that respond to an empirical therapy approach may never be cultured, and therefore the spectrum of submitted samples is likely biased toward more resistant infections. These may potentially include cases of repeated or ongoing infection in which multiple therapies have been tried and failed before susceptibility testing was conducted. Although well-intentioned to inform veterinarians on the samples received at the laboratory, publishing this type of data without appropriate context could unintentionally misguide treatment decisions in routine cases.

The collation and analysis of laboratory results are most useful when interpreted in conjunction with submission information. However, laboratory submission forms frequently lack information that is considered necessary for disease detection, analysis, and reporting, such as history, geographic location, farm type, group type, and the number of animals that are at risk, sick, and dead.^7^ Cases lacking premises identification numbers make it difficult to determine if multiple cases have been submitted from a single premises. When working with a small number of test results, the ability to group submissions by premises is particularly critical because multiple submissions from a single premises experiencing a disease outbreak can falsely be interpreted as a more widespread increase in disease. Another important data element for assessing risk is commodity, also known as animal industry type. For example, within the poultry network, data are assessed separately for layers, broilers, turkeys, and small flocks, and submissions lacking this information cannot be fully evaluated.

Another challenge when examining surveillance data is that a precise denominator is rarely known for the population being assessed. Typically, only count data for positive tests are available; however, without a denominator, there is no measure of the size of the population (which may be changing over time) from which the cases arose. Generally, endemic diseases have a stable case count with little fluctuation and tend to be of lower interest to networks. Ideally, if a disease is increasing or the pattern is changing, this should be verified before additional prevention or control steps are recommended. It is possible that an observed increase in disease may be the result of how surveillance was conducted rather than a true change in disease occurrence. There are a multitude of reasons for an artifactual change such as the availability of a new test or an increase in veterinarian awareness and test selection, or market variability that affects decisions by veterinarians and producers to submit diagnostic samples. An ongoing challenge for all networks is lag time—by the time the cases for the quarter are compiled and reports from networks are issued, the information can be out-of-date.

When reviewing data, network members do their best to evaluate how laboratory data align with other streams of information submitted to the networks. Clinical impression surveys may be prone to recency bias in which veterinarians are more likely to remember and report cases examined in the more immediate past compared to the beginning of the time period. Laboratory data summarized over the same quarter do not have the same bias, and all forms of information can serve as a check and balance for each other.

### Network actions to improve the number and quality of laboratory submissions

With such a dependence on laboratory results to support surveillance, there is a need to reduce laboratory errors as much as possible, particularly preanalytical errors.^11^ Unsuitable samples as a result of misidentification, quantity (insufficient volume to perform the analysis, inadequate blood-to-anticoagulant ratio), or quality (hemolyzed, lipemic, samples in the wrong container) issues represent the majority of preanalytical problems, which account for up to 70% of total laboratory errors.^9^ Part of the response to reduce these errors is through education for those submitting samples. One of the main goals of the OAHN is to provide a communication platform for industry with a focus on veterinarian education. As a response to support improved sample submission, OAHN networks have produced infographics for veterinarians on the “do’s and don’ts” of laboratory sample submission, such as using the appropriate containers for the desired sample and using digital submissions to avoid errors related to illegible forms. Social media platforms were also used to communicate the message of appropriate sample submission to the veterinary community online. Discussions of appropriate sample submission as well as appropriate test selection are presented as part of disease discussions in the individual communications products, such as reports and podcasts produced by the networks.

For some species groups, obtaining a sufficient number of laboratory samples is a hindrance to developing a surveillance program or to observe trends. Three networks, the small ruminant, aquatic animal, and bovine networks, addressed this issue through OAHN-funded projects. The small ruminant network used an online platform to encourage sample submissions from veterinarians investigating adult sheep and goat mortalities on Ontario farms.^14^ A website was developed for veterinarians to submit history, clinical and postmortem findings, and digital postmortem photographs. A complete set of formalin-fixed and fresh tissues were sent to the AHL for comprehensive diagnostic testing. As a result of the project, causes of morbidity and mortality were diagnosed more frequently, thus laboratory testing was perceived as a more valuable practice within the industry and better management decisions were made. A useful product developed from this project was a laminated postmortem template used by practicing veterinarians to guide the amount of sample to submit from necessary organs and tissues. The network also developed 2 whiteboard videos for producers entitled: “The value of a postmortem for your sheep flock/goat herd.”

The bovine network also conducted a postmortem project aimed at promoting sample submission.^5^ The objective of the project was to provide funding to veterinarians to conduct more calf postmortem examinations to support disease intelligence at the herd level, with an additional benefit of improving surveillance for *Salmonella enterica* serovar Dublin. The outcomes of this project included redirecting on-farm investigations, changing or minimizing therapies, promoting more laboratory testing, identifying zoonotic infections and risk factors, and encouraging preventive practices by producers.

In another project to promote sample submission, the aquatic animal network funded aquaculture veterinarians to submit fish from Ontario farms to improve surveillance of bacterial pathogens and antibiotic-resistant strains of bacteria.^4^ Aquaculture in Ontario has typically not had a large veterinary presence and, prior to recent regulatory changes, antimicrobials had been utilized without a veterinary prescription. Therefore, this project’s objective was to identify common aquatic animal pathogens in Ontario and profile antimicrobial resistance among them. The subsidized testing resulted in more frequent testing by producers and veterinarians, which resulted in the detection of pathogens or diseases that could have otherwise been misdiagnosed, such as epitheliocystis, and promoted more prudent antimicrobial usage. An important benefit of this project was to assist in the establishment of veterinarian–client–patient relationships in the aquaculture industry where none had existed previously. The resultant effect was to demonstrate to farm operators the value of sample submission and antimicrobial susceptibility testing using minimum inhibitory concentrations to decrease the use of therapeutics in food fish, track resistance to therapeutics, and adapt treatment protocols.

Sometimes attempts to increase sample submission go hand-in-hand with test development, particularly for a species for which few tests exist, and therefore laboratory surveillance data is lacking. American foulbrood (AFB), a devastating bacterial brood disease of honeybees (*Apis mellifera*), is caused by ingestion of spores of *Paenibacillus larvae* by honeybee larvae within 12 to 36 h of hatching. Previously, there was little information on AFB and its causative agent *P. larvae* in Ontario when only clinical cases were submitted for culture and susceptibility testing at the USDA Bee Research Laboratory (Beltsville, MD). When the USDA laboratory stopped accepting Ontario samples in 2015, the Ontario bee industry needed another laboratory to provide bacterial culture of *P. larvae* for the monitoring of susceptibility of *P. larvae* to antimicrobials used for prevention. The OAHN bee network sponsored a project to collect samples from both symptomatic and asymptomatic colonies in conjunction with detailed colony examination by specialists, culture Ontario *P. larvae* isolates, and identify them using spectra present in the matrix-assisted laser desorption/ionization time-of-flight mass spectrometry (MALDI-TOF MS) biotype database.^13^ This testing is now available as a part of the routine testing service offered by the AHL, and it has been used to test honey samples exported worldwide.

### Discoveries in animal health resulting from network surveillance

Disease intelligence obtained from network discussions of laboratory data and clinical impressions can lead to exciting discoveries in animal disease, as well as drive further surveillance. An OAHN equine project investigating the detection of *Neorickettsia risticii*, the causative agent of Potomac horse fever (PHF), was prompted by frustration among practicing veterinarians who treated horses with clinical signs consistent with PHF but for which testing proved negative. Moreover, there were discrepant results between sample types (feces vs. blood), tests (PCR vs. culture), and diagnostic laboratories. The network investigated the performance of 2 diagnostic laboratories for molecular detection of *N. risticii* in blood and fecal samples from horses with clinical signs consistent with PHF, and found excellent agreement between laboratories in the ability to detect *N. risticii* nucleic acid in fecal samples, but not in blood. From this project, both blood and fecal samples proved adequate for molecular detection of PHF, but there may be discrepancies between laboratories based on sample type. Additionally, the project detected a novel *Neorickettsia* species (*Neorickettsia findlayensis* sp. nov.) that tested negative on existing PHF PCR testing.^16^

The small ruminant network set out to determine the seroprevalence of *Toxoplasma gondii*, a zoonotic infectious agent in sheep and goats in Ontario herds. A cross-sectional serologic survey of sheep and goat farms was conducted between August 2010 and February 2012, and sera were analyzed using an immunofluorescent antibody test (IFAT).^10^ High seroprevalence was identified among farms, indicating that there is a risk to humans of contracting infection from *T. gondii* that may occur from consumption of undercooked meat or unpasteurized milk. It also suggested that the risk of abortion and neonatal loss caused by *T. gondii* infection is high in Ontario flocks and herds. As a follow-up project in 2018, the small ruminant network funded the validation of a *T. gondii* real-time PCR test that has higher sensitivity for detecting protozoal infection and would assist in detecting the etiologic agent of small ruminant abortion cases.^15^ To alert small ruminant veterinarians and producers, an information sheet on toxoplasmosis in sheep and goats was also produced.

Network discussion surrounding 2 cases of canine brucellosis (*Brucella canis*) prompted the companion animal network to undertake a project investigating the seroprevalence of *B. canis* in commercial dog-breeding kennels in southwestern Ontario.^18^ Overall, *B. canis* was identified serologically in 127 of 1,056 (12%) dogs from 22 of 64 (34%) kennels. The prevalence at the kennel level was 0–100%. Serial testing was performed on a subset of dogs, and 27 dogs with reactive results were followed. Twenty-four dogs became negative on subsequent testing, consistent with transient cross-reaction as can be found in situations such as following *Bordetella bronchiseptica* vaccination. Two other dogs remained reactive and one seroconverted. These findings have important ramifications for dealing with reactive test results, highlighting the potential for false-positives. Further work in this area will involve continued communication with kennels to try to reduce *B. canis* in the breeding dog population. Education of physicians, public health personnel, and veterinarians is ongoing to increase awareness of *B. canis* infection and disease.

Another project looked at the risk of chronic wasting disease (CWD) in Ontario. The range of white-tailed deer in Ontario has been expanding, and as a big-game species, they harbor important zoonotic or potentially zoonotic diseases. CWD is a transmissible spongiform encephalopathy that has been detected in nearly all jurisdictions bordering Ontario. Variation at the prion protein (*PRNP*) gene causes a variation in how quickly deer display signs of CWD and how long they shed prions into the environment, potentially influencing the rate and nature of the spread of CWD. The OAHN wildlife network supported research to characterize the prevalence and spatial pattern of the *PRNP* gene to inform surveillance and monitoring of CWD in Ontario.^12^ A total of 631 samples from yearly CWD surveillance of hunter-harvested wild deer were obtained from the Ontario Ministry of Natural Resources and Fisheries repository. There were no significant differences in the presence of the variants of the *PRNP* gene across geographical areas, and numerous previously unidentified alleles were found. This information forms a baseline for further work and can be used to assess the natural gene flow of white-tailed deer in Ontario and simulate the most likely pattern of CWD spread through the province if CWD is detected. As a follow-up to the report posted on the OAHN wildlife webpage, a podcast was also produced explaining the project and its outcomes.

Often disease intelligence from a variety of sources drives disease investigation. For the poultry and swine networks, condemnation reports as well as observations from abattoirs provide additional information to support disease trends. The poultry network discussed reports of increasing cases of proventricular dilation occurring initially at processing plants and subsequently from veterinarians in the broiler industry. Pathologists at the AHL also confirmed an increase in poultry submissions with proventricular lesions resembling transmissible viral proventriculitis (TVP). Affected birds were full of feed (up to 300–400 g) at processing and there was no feed passage after being returned to lairage, leading to increased condemnations as a result of contamination. The proventricular dilation problem at processing plants seemed to wax and wane during the year, but continued to be present in reports from veterinarians of higher on-farm mortality because of sudden death, as well as pendulous crops and feed regurgitation. Samples of proventriculi from affected flocks on-farm and at processors were sent for immunohistochemical staining (IHC) for chicken proventricular necrosis virus (CPNV), the etiology of TVP.^3^ The results indicated that TVP is present in Ontario broilers, but to a limited degree, and that further investigation is warranted into the cause of proventricular dilation syndrome in Ontario.

Similarly, the swine network noted an increase in swine erysipelas based on data from the quarterly clinical impressions survey, as well as data from provincial and federal abattoirs, but found no corresponding increase in laboratory data. The network proceeded to investigate the disease and characterize isolates from swine erysipelas cases from abattoirs and swine farms in Ontario. Tissue samples (spleen and lung) collected from hogs at abattoirs as well as clinical cases in Ontario with lesions suspicious for swine erysipelas were submitted to the AHL for culture. Eleven samples were collected from clinical cases and 14 samples from abattoirs. Only 6 isolates (from 3 clinical cases) of *Erysipelothrix rhusiopathiae* were recovered (unpub. data). Given the low number of isolates, archived isolates from the AHL (*n* = 5) and Gallant Custom Laboratories (*n* = 3) were also included to provide a total of 14 isolates for sequencing. Whole-genome sequence (WGS) data were used to detect antimicrobial resistance genes, virulence genes, and to establish multi-locus sequence typing (MLST) of Ontario isolates. The results established a WGS database of 14 isolates that can be expanded by adding the WGS of new isolates to monitor their epidemiologic relatedness and to detect the presence of resistance genes.

### Communication of laboratory data and network findings

**Figure 3.**
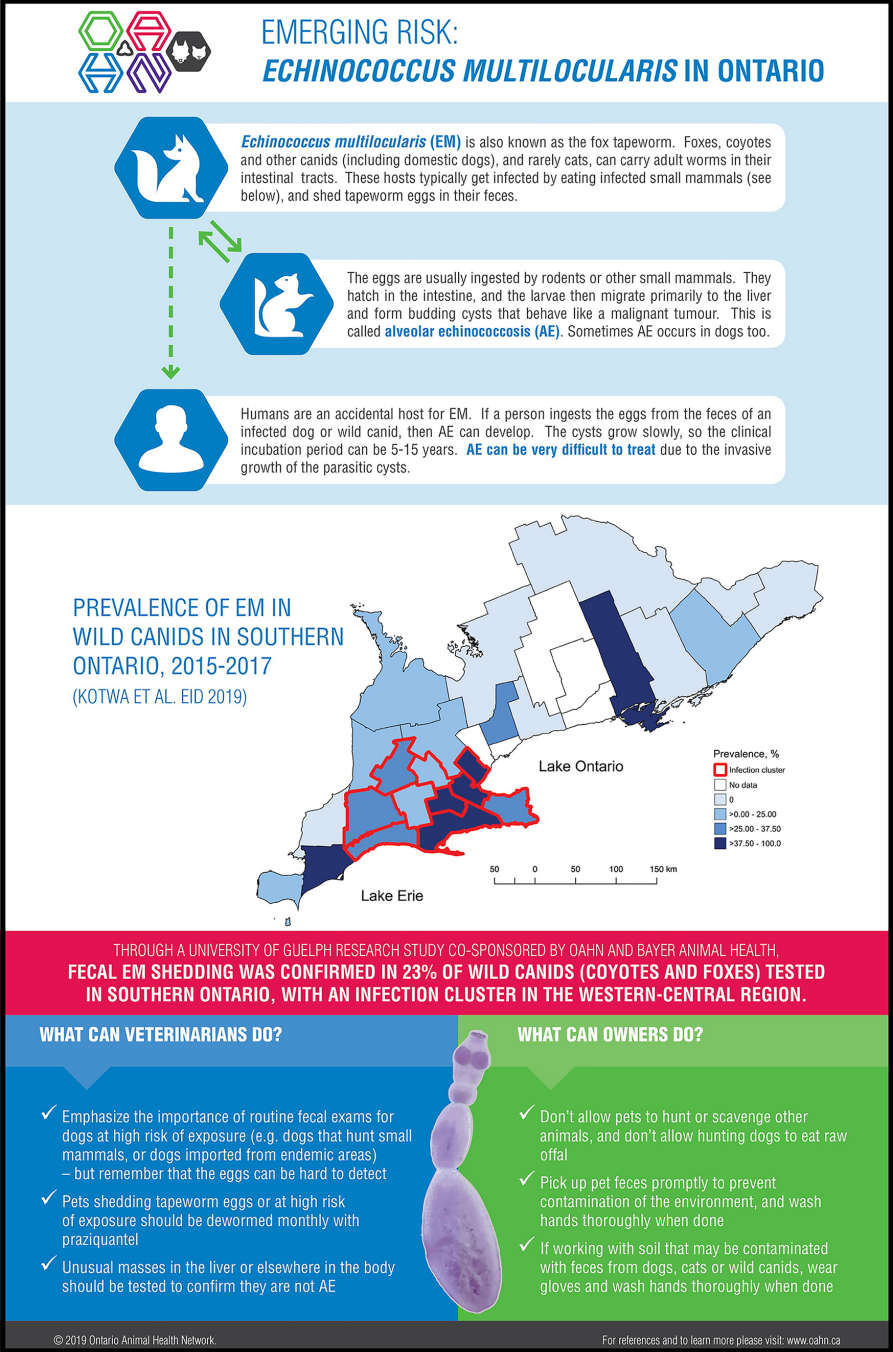
An infographic produced by the companion animal network of the Ontario Animal Health Network to communicate information on the emerging risk of *Echinococcus multilocularis*.

Laboratory data and activities of the networks are communicated to veterinary practitioners and industry through a variety of means. Regular veterinary and industry reports are compiled, typically each quarter, that summarize the pertinent information for end-users. Messaging laboratory data is approached by summarizing trends seen over the time period and highlighting unique or emergent cases. Individual networks most often use a written report but have also used podcasts and industry magazine articles to communicate surveillance findings. Where laboratory data surveillance has generated disease investigation or research projects, these have been disseminated to veterinarians and industry members as written reports, infographics, posters, podcasts, and videos. Social media accounts trigger awareness for followers when new information is available; growing on-line engagement with network products is monitored to support future communication strategies. For example, the companion animal network realized the benefit of infographics for disseminating information on leptospirosis and *Echinococcus multilocularis* infection (Fig. 3) and have continued using that communication method for other topics. The swine network felt engagement was poor on Twitter because swine tweets were lost among information regarding other species. To address this, a dedicated swine network Twitter account was created to focus solely on commercial swine industry health information, and engagement has increased.

Network products are shared with other provincial surveillance networks such as RAIZO and are also shared with national surveillance networks such as the Canadian Animal Health Surveillance System (CAHSS) and the Canadian Swine Health Intelligence Network (CSHIN). Information sharing between surveillance networks allows each species network to benefit from the findings and analysis in other provinces. The benefit of a provincial surveillance system is limited without a national animal disease surveillance system. Through coordination of information sharing, disease management, and responses, a national animal health and disease surveillance system can help prevent the introduction and spread of disease across geographic boundaries, thus safeguarding national animal health, welfare, and food safety.

## Conclusion

Since its inception in 2013, the OAHN has been focused on creating a collaborative, cross-species web to utilize available data for the improvement of animal health in the province. The network structure is similar across all species but allows for flexibility to customize for each sector’s needs. Mining data from veterinary laboratories and collating it into useful visualizations for the networks is a key operation of the pathology and coordination team. Multiple data sources, including data from other laboratories, provincial abattoirs, surveys from veterinarians in the sector, and other disease reports are collated for human analysis. However, the data have limitations, and care must be taken not to over-interpret. The addition of targeted research projects has allowed networks to investigate, confirm, or refute trends noted in the data review process. In many instances, these projects have provided information that helps clinicians in the province make more knowledgeable decisions about challenging cases, and in one occurrence, has led to the discovery of a novel pathogen. The networks also offer another channel between the laboratory and clinicians to collaborate to improve sample submission quality and transfer knowledge about infectious disease. The networks continue to evolve and adapt to the needs of each sector and the concerns of the day, focusing on using multiple data sources and human-based review to build upon the knowledge and trust flowing among practitioners, producers and owners, academia, and government.
